# Modelling the spread of bovine viral diarrhea virus (BVDV) in a beef cattle herd and its impact on herd productivity

**DOI:** 10.1186/s13567-015-0145-8

**Published:** 2015-02-24

**Authors:** Alix Damman, Anne-France Viet, Sandie Arnoux, Marie-Claude Guerrier-Chatellet, Etienne Petit, Pauline Ezanno

**Affiliations:** INRA, UMR1300 BioEpAR, CS 40706, F-44307 Nantes, France; Oniris, LUNAM Université, UMR BioEpAR, F-44307 Nantes, France; FRGDS Bourgogne, F-21000 Dijon, France

## Abstract

Bovine viral diarrhea virus (BVDV) is a common pathogen of cattle herds that causes economic losses due to reproductive disorders in breeding cattle and increased morbidity and mortality amongst infected calves. Our objective was to evaluate the impact of BVDV spread on the productivity of a beef cow-calf herd using a stochastic model in discrete time that accounted for (1) the difference in transmission rates when animals are housed indoors versus grazing on pasture, (2) the external risk of disease introductions through fenceline contact with neighboring herds and the purchase of infected cattle, and (3) the risk of individual pregnant cattle generating persistently infected (PI) calves based on their stage in gestation. The model predicted the highest losses from BVDV during the first 3 years after disease was introduced into a naive herd. During the endemic phase, the impact of BVDV on the yearly herd productivity was much lower due to herd immunity. However, cumulative losses over 10 years in an endemic situation greatly surpassed the losses that occurred during the acute phase. A sensitivity analysis of key model parameters revealed that herd size, the duration of breeding, grazing, and selling periods, renewal rate of breeding females, and the level of numerical productivity expected by the farmer had a significant influence on the predicted losses. This model provides a valuable framework for evaluating the impact of BVDV and the efficacy of different control strategies in beef cow-calf herds.

## Introduction

Bovine viral diarrhea virus (BVDV) affects most industrialized cattle farming systems by inducing reproductive disorders (abortion, delayed calving, reduced fertility) in breeding cattle and by lowering herd productivity through increased culling, morbidity, and mortality [1]. Introductions may occur through the direct purchase of infected animals when cattle are housed indoors as well as through fenceline contact with infected animals in neighboring herds when cattle are grazed outdoors on pasture. The likelihood of these introductions depends on the control measures that are implemented by individual farms. In some areas (e.g. Brittany, France: [2]), purchased animals are guaranteed not to be persistently infected based on knowledge of their dam status, previous diagnostic testing, or their source herd status. In other areas, although there are pre-purchase diagnostic tests available for BVDV, most farmers tend not to use them [3,4]. It is also difficult to determine whether pregnant females on pasture are at subsequent risk of delivering persistently infected (PI) calves since there are few reliable prenatal tests for BVDV. The severity of production losses following disease introduction is also related to several additional management factors, including (1) the level of herd immunity from previous natural exposure [5] or preventative vaccination [6], (2) the percentage of dams that are at risk for generating PI calves through vertical transmission, and (3) the ability for BVDV to spread within and between different production subgroups within a herd [7]. In the absence of a calf surveillance scheme, it may be difficult for farmers to detect the presence of BVDV in the herd leading to the establishment of an endemic disease state and long term production losses.

Modelling is a pertinent approach to predict pathogen spread and persistence in a herd and to evaluate its impact on herd dynamics and productivity for a large range of management scenarios [8]. Many of the modelling studies conducted to date have concerned dairy cattle herds, both at the herd [7,9,10] and regional scales [11-13]. However, beef cow-calf herds have a unique demographic structure that has not been captured by previously published models. First, beef cattle are frequently grazed outside for long periods, particularly at the time when pregnant dams have the greatest risk of generating PI calves following exposure to BVDV through fenceline contacts. Second, the calving period is concentrated over a few months and calves are raised with cows until weaning. This increases the duration and intensity of exposure to BVDV as PI animals mainly are observed in young stock due to a shortened life expectancy [14]. Therefore, conclusions drawn for dairy herds cannot be directly transferred to beef farming systems. Models of BVDV spread in a beef herd have been proposed to evaluate the costs associated with epidemics in naive herds [15] or to compare control [16] and testing [17] strategies. Two recent models account for BVDV introduction due to animal purchases or fenceline contacts [5,18], representing an endemic situation. However, none of these models simultaneously account for the within-herd contact structure, the difference between the indoor and outdoor periods in within-herd virus transmission, and the risk of continuous virus introduction due to the purchase of animals and contacts with neighboring infected herds. All of these processes are expected to greatly influence BVDV spread and persistence in a beef cow-calf herd and, consequently, impact the associated losses.

Our objective was to evaluate the impact of BVDV spread on the productivity of a beef cow-calf herd across a large range of management scenarios. A stochastic epidemiological model was proposed that accounted for different transmission rates between separately managed production groups during the outdoor grazing period versus a homogeneous population structure during the indoor period. The model also incorporated an external risk of BVDV introduction through fenceline contacts with neighboring herds as well as through animal purchases. A sensitivity analysis was conducted to determine the relative importance of management factors such as herd size, the length of breeding, grazing, and selling periods, the replacement rate of breeding females, and level of numerical productivity expected by the farmer. Comparisons of production losses in acute outbreaks and endemic situations were also performed.

## Materials and methods

A stochastic compartmental model in discrete time was developed to simulate the spread of BVDV. A time interval of 7 days was chosen as the longest as possible to properly represent transiently-infected animals in the infection process. The model was fully implemented in C++, allowing the model to be run rapidly.

### Herd dynamics

**Figure 1:**
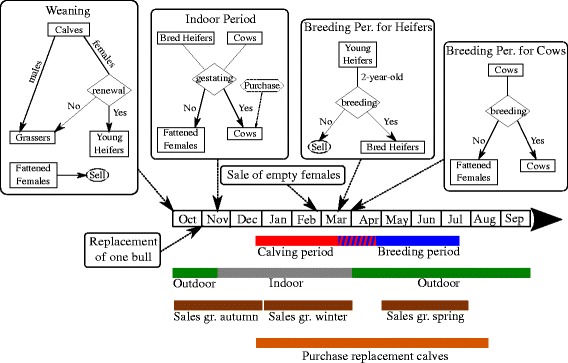
**Diagram of the beef cow**-**calf herd dynamics over a year.** Each box represents the between-group transitions at a given time in the year. A diamond symbolizes a selection process. Groups are the following: calves from birth until weaning, male and female weaned calves for sale (grassers), heifers younger than 2 years kept for breeding, bred heifers, cows from the first pregnancy diagnosis until fattening decision, cows from fattening decision until culling, and bulls. Calving, breeding, grazing, and purchasing/selling periods are indicated and correspond to the reference scenario as described in section Initial conditions, reference scenario, and simulations.

The farming system modelled and used for simulations in this work was based on the characteristics of beef cow-calf herds in Bourgogne, one of the main beef production regions in France. In this area, animals are indoors during the winter and most of them are outdoors from spring to autumn (Figure 1). Purchases are limited over time and consist of replacement calves, pregnant females, and bulls for replacement. The herd dynamics presented here was based on that presented in [19].

The number of females kept for breeding was fixed according to the target number of calves weaned per year, adjusted according to the anticipated losses from routine infertility and calf mortality. Anticipated losses varied according to the level of numerical productivity expected by the farmer, stated by the adjustment factor *ε* (Table 1).

**Table 1:** **Definitions and values of model parameters**

**Parameters**	**Values**	**Definitions**	**Sources**
*ρ*_*sex*_	0.5	Sex ratio	
*a*, *b*	8.7, 6	Parameters of the gamma distribution used to calculate the next start of pregnancy	
*δ*	0.035	Probability of twin birth	
*τ*_*He*_	0.02	Probability of infertility for heifers	
*τ*_*Co*_	0.08	Probability of infertility for cows	
*breeding*_*start*	15^th^ of March	Date of start of the breeding period	
*breeding*_*dur*	[16 **18** 20]	Duration of the breeding period (in weeks)	
*weaning*	1^st^ October	Date of weaning	
*pasture*_*start*	1^st^ April	Date of start of the pasture period	
*pasture*_*dur*	[29 **32** 35]	Duration of the pasture period (in weeks)	
*renewal*_*rate*	[0.286 **0.317** 0.349]	Ratio heifers/cows	
*sell*_*period*	[autumn **winter** spring]	Sell period of grassers^1^	
*size*	[42 **83** 125]	Number of bred females^2^	
*μ*_*Ca*,*bi*_	0.0225	Probability of mortality at birth of calves	
*μ*_*Ca*_	0.000333	Mortality rate of calves (d^-1^)	
*intro*_*week*	[(20 25 30) **27** 40]	Week of introduction of PI animal(s)^3^.	
*ε*	[0.95 **1** 1.05]	Level of numerical productivity expected by the farmer	
*μ*_*P*,*bi*_	[0.06 **0.0667** 0.0733]	Probability of mortality at birth of PI calves	
*μ*_*P*_	[0.0017 **0.0019** 0.0021]	Mortity of PI animals per day	[20]
*ϕ*_*MS*_	[0.006 **0.00667** 0.00733]	Trantion rate from state *M* to state *S* (d^-1^)	[21]
*ϕ*_*TR*_	[0.18 **0.2** 0.22]	Transition rate from state *T* to state *R* (d^-1^)	[22]
*β* ^*T*^	[0.027 **0.03** 0.033]	Daily transmission rate for *T* animals	[9,23]
*β* ^*P*^	[0.45 **0.5** 0.55]	Daily transmission rate for PI animals	[9,24]
${\beta}_b^P$	[0.09 **0.1** 0.11]	Daily between-group transmission rate for PI animals	[9,25,26]
*α*_*Ra*_	[0.72 **0.8** 0.88]	Abortion rate due to infection in early pregnancy	[22,27]
*α*_*Rb*_	[0.18 **0.2** 0.22]	Abortion rate due to infection in mid-pregnancy	[28,29]
*η*_*X*_		Probability of giving birth to a calf in state *X* if infection in mid-pregnancy and no abortion	[24,28-30]
*η*_*P*_	[0.875 **0.9375** 1]		
*η*_*M*_	[0.0625 **0.03125** 0]		
*η*_*R*_	[0.0625 **0.03125** 0]		
*K*_*ext*_	0	Risk of virus introduction on pasture	

Nominal values are in bold. Other values are the ones tested in the model sensitivity analysis.

^1^In the reference scenario, grassers were sold at the three periods: 45% in autumn, 45% in winter, and 10% in spring.

^2^Bred females are heifers and cows, with: 42 = 10 heifers + 32 cows, 83 = 20 heifers + 63 cows, 125 = 30 heifers + 95 cows.

^3^In the first case, 3 PI animals were introduced successively in weeks 20, 25, and 30. In the two other cases, a single PI animal was introduced.

The herd was structured into 7 groups: calves from birth until weaning, male and female weaned calves for selling (grassers), heifers under the age of two kept for renewal, bred heifers, cows from the first pregnancy diagnosis until fattening decision, cows from fattening decision until culling, and bulls. The herd dynamics relies on specific dates when animals change groups (Figure 1; Table 1).

At weaning, some female calves were grouped with young heifers for renewal while others were fattened for sale during the year. The gender of calves was determined stochastically according to the sex ratio *ρ*_*sex*_ (Table 1). The number of females selected for renewal was fixed. In case of unexpectedly high calf mortality, replacement calves could be purchased. The replacement of a dead calf was allowed from the beginning of the calving period until three months after its end. Purchase occurred if the number of calves present and to be born in the herd fell below the production objective.

At the beginning of the indoor period, all pregnant heifers and cows were merged to be raised together while non-pregnant ones were fattened for 100 days before being sold. The model determines the expected number of calvings according to the production objective. If the expected number of pregnant animals was below the target number of calvings to meet production objectives, pregnant females were purchased at the beginning of the indoor period to reach this number.

At the beginning of the breeding period, a fixed number of females was selected among the 2-year-old heifers to form the group of bred heifers. The remaining 2-year-old heifers were sold. Cows were split into two groups. A fixed number of cows formed the breeding stock while the others were fattened until their calves were weaned. Then, they were culled. The period began 2 weeks earlier for heifers than for cows, and ended when bulls were separated from breeding females. We integrated in the compartmental model an individual-based monitoring of pregnant females. From the start of pregnancy until calving, each female is represented individually to precisely predict her stage of pregnancy over time.

Calving occurred 285 days after the beginning of gestation and the mother was then not available for breeding for a period of 20 days. Twins were born with probability *δ* (Table 1). During the breeding period, the delay between the moment when a cow was available for breeding and the start of a new pregnancy was determined by a gamma distribution with parameters *a* and *b*. The values of these parameters (Table 1) were chosen to reproduce the observed calving-to-calving intervals as presented in [19]. The same gamma distribution with the same parameter values was used regardless of whether breeding females were indoors or at pasture. The date of a new pregnancy was calculated if the animal was not declared infertile. The probability of infertility of heifers and cows is given by parameters *τ*_*He*_ and *τ*_*Co*_, respectively (Table 1). The calculated new pregnancy date must be before the end of the breeding period otherwise the animal was considered as non-pregnant.

The simulated average date of calving was 60 days after the beginning of the calving period. The indoor and breeding periods can be chosen within a certain range. It was assumed that both the calving and the breeding periods started during the indoor period. The date of weaning was chosen so that calves were weaned at the average age of 6 up to 8 months on the field.

All animals placed in the group of grassers after weaning were sold over the course of the year. The model simulates various periods for sales. These periods were specifically considered because they impact the duration that potentially infected animals are present in the herd. Indeed, animals can be sold at one, two or three periods in the year. Dates of selling were randomly chosen each year using triangular distributions for the three periods: (23/10, 15/11, 07/12), (23/01, 15/02, 07/03) and (23/05, 15/06, 07/07). The proportion of animals sold at each period was fixed at the beginning of each simulation.

The number of bulls present in the herd was assumed to be constant. The model assigns one bull per 20 bred heifers or cows. Each year, on November 1st, a bull was randomly selected for replacement.

### Within-herd infection dynamics

**Figure 2:**
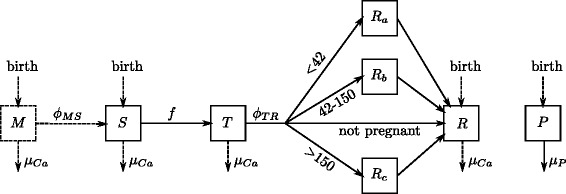
**Diagram of the transitions between health states.** *S*: susceptible; *T*: transiently-infected; *P*: persistently infected; *M*: protected by maternal antibodies; *R*: immune; *R*_*a*_, *R*_*b*_, *R*_*c*_: immune which have been infected in early, mid and late pregnancy, respectively. Dotted lines concern only calves. The values of the transition rates *ϕ*_*MS*_ and *ϕ*_*TR*_ are given in Table 1. The value of the transition rate f is derived from eq. 1 and eq. 2.

Animals were classified into mutually exclusive BVDV health states (Figure 2): susceptible (*S*), transiently-infected (*T*), recovered, i.e. immune (*R*), protected by maternal antibodies (*M*) or persistently infected (PI; *P*). The *M* to *S* and *T* to *R* transitions depend on transition rates *ϕ*_*MS*_ and *ϕ*_*TR*_ (Table 1). Since maternal protection generally lasts 4-6 months [31], we assumed that the M to S transition occurred only in the Calf group. The *S* to *T* transition represents horizontal transmission. It depends on the repartition of the shedding animals (*T*, PI) in the herd.

During the indoor winter period, the risk of infection is assumed to be equally distributed among all animals. In that case, the transmission rate *f* is given by

1 $$ f={\beta}^P\frac{N^P}{N}+{\beta}^T\frac{N^T}{N}, $$

where *N*^*P*^ and *N*^*T*^ are the total numbers of PI and *T* animals in the herd, respectively; *N* the herd size, and *β*^*P*^ and *β*^*T*^ the transmission rates per day associated with the PI and *T* animals, respectively (see Table 1).

During the outdoor period, animals were split into separate pastures except remaining grassers which remain indoors until they were sold. Three groups were considered to be raised on different pastures: young heifers, bred heifers plus part of the bulls, and cows (with their calves) plus remaining bulls. We assumed this latter group to be homogeneously mixed. Virus transmission outdoors was due to transmission within each group (with the same formulation as indoors) and between pairs of groups (as in [7], accounting for the size of both groups in contact and for PI animals only as virus sources). Contacts with PI animals from neighboring farms may also occur. Since this risk is unknown, we assumed here a constant risk *K*_*ext*_. Hence, transmission rate *f*_*k*_ associated with animals in pasture *k* reads:

2 $$ {f}_k={\beta}^P\frac{N_k^P}{N_k}+{\beta}^T\frac{N_k^T}{N_k}+{\beta}_b^P\left(\frac{K_{ext}}{N_k}+{\displaystyle \sum_{k\hbox{'}\ne k}}\frac{N_{k\hbox{'}}^P}{N_{k\hbox{'}}{N}_k}\right), $$

with ${N}_k^P$ and ${N}_k^T$ the numbers of PI and T animals in pasture *k*, respectively, *N*_*k*_ the number of animals in pasture *k*, *k*’ all the pastures in contact with pasture *k*, and ${\beta}_b^P$ the between-group transmission rate per day associated with PI animals.

Immune cows (*R*) gave birth to calves protected by maternal antibodies (*M*) acquired via colostrum. Susceptible (*S*) or PI (*P*) cows gave birth to calves in the same state. If mothers were infected during pregnancy, consequences differed depending on the stage of the pregnancy at the time of infection. As females are individually monitored during pregnancy, their stages are known precisely. The pregnancy period was divided into three stages: early pregnancy (0-41 days; *R*_*a*_), mid pregnancy (42-150 days; *R*_*b*_), and late pregnancy (151-285 days; *R*_*c*_). Infection during the first stage led to either embryonic or fetal death with probability *α*_*Ra*_ (Table 1), or the birth of a calf protected by maternal antibodies (*M*). Different consequences of infection during mid-pregnancy are possible [22,24,27-30]. The pregnant female may abort with probability *α*_*Rb*_ (Table 1). If not, the calf can be born in states *M*, *R*, or *P* with probabilities *η*_*M*_, *η*_*R*_, or *η*_*P*_, respectively. Finally, if infection occurs during late pregnancy, the mother gives birth to an immune calf (*R*). In case of abortion, a delay of 60 days is applied between the end of infection and abortion. The female is then unavailable for breeding for 20 days. If abortion occurs not too late, i.e. at least 20 days before the end of the breeding period, the female may return to the pregnancy state using the algorithm explained in the previous section.

All transitions between health states as well as births and deaths are schematically represented in Figure 2. Transition rates per day are given in Table 1. Transfers between health states were performed using binomials. The probability of transition from compartment *i* to *j* is given by

3 $$ {p}_{ij}=1- exp\left(-\varDelta t{\tau}_{ij}\right), $$

where *Δt* is the time step (7 days) and *τ*_*ij*_ the daily transition rate between compartments *i* and *j*. The transition *ΔN*_*ij*_ is then calculated as

4 $$ \varDelta {N}_{ij}= Bin\left({N}_i,{p}_{ij}\right), $$

where *N*_*i*_ represents the number of individuals present in compartment *i*. In the case of multiple transfers, we used multinomials instead [32]. The transfers between groups were made by randomly selecting animals from all health states.

Females in the group of young heifers stayed two years in that group. For this group, all compartments were doubled to differentiate one-year and two-year-old heifers. At the beginning of the reproduction period, only two-year-old heifers were either selected for breeding and transferred in the bred heifer group, or sold.

At birth, PI and non-PI calves had a probability of dying of *μ*_*P*,*bi*_ and *μ*_*Ca*,*bi*_, respectively (Table 1). The proportion of deaths at birth for PI calves encompassed the deaths of abnormal calves. The model associates a mortality rate *μ*_*Ca*_ with non-PI calves between calving and weaning and *μ*_*P*_ with all PI animals (Table 1). Finally, 9% of all calves died before weaning and PI animals had a half-life of 1 year.

Purchased animals (calves, pregnant females, and bulls) can be of any health state in the model (*S*, *T*, *P*, *RP*).

### Initial conditions, reference scenario, and simulations

A simulation year started after weaning (1^st^ October), corresponding to week 0 (Figure 1). In the reference scenario, the indoor period ranged from week 6 to 26 (mid-November to March). The breeding period started during the indoor period on week 23 (mid-March) for bred heifers and on week 25 (end of March) for cows. It finished on week 41 (mid-July) when bulls were separated from breeding females. The calving period ranged from week 12 to 29. It corresponds to the indoor period plus one week. The initial herd was obtained by running the model for 4 years without BVDV introduction. The fourth year was used to obtained mean reference values for purchases, sales, and number of weaned calves. Then, the birth of a PI calf was simulated at the beginning of the cow breeding period (week 27) in an average herd representative of herds in the Bourgogne region (France). The number of bred heifers and cows are 20 and 63, respectively. We assumed a basic level of numerical productivity expected by the farmer, i.e. the farmer does not expect losses to differ from usual infertility of breeding females and calf mortality (ε = 1). In such a situation, the production objective was equal to 73 weaned calves per year (for 83 bred females). After introducing a PI animal, the simulation continued for 15 years, the first three years being representative of an acute phase (infection arising in a naive herd), whereas years 6 to 15 were representative of an endemic phase (when infection persists in the herd). In the reference scenario, we assumed that all the purchased animals were susceptible and that no infection due to neighboring contacts occurred (*K*_*ext*_ = 0). For each scenario considered thereafter, 3000 repetitions were performed.

### Outputs

Outputs were selected to represent infection dynamics and the impact of BVDV on herd productivity. Outputs associated with infection dynamics are the probability of virus persistence in the herd (infected herds having ≥ 1 PI or T animal, or ≥ 1 immune dam carrying a PI fetus), and the prevalence of PI and T animals and of immune dams carrying a PI fetus (state RP). Prevalence of PI and T animals represents the proportion of PI and T animals in the whole herd while prevalence of immune dams carrying a PI fetus is restricted to breeding females only. Outputs related to herd productivity are the number of losses (abortions and deaths of PI animals), purchases (replacement calves, pregnant females and bulls), weaned calves, sales of grassers and young heifers, and sales of empty and fattened females. To evaluate the impact of BVDV on herd productivity, we subtracted the contribution of the reference year (the last year before the first BVDV introduction) from these last outputs, considering only the relative change with and without BVDV circulating. Losses and purchases were also evaluated per bred female to remove the direct impact of herd size on such outputs.

For all outputs except virus persistence, we calculated the annual median value with an 80% credible interval (P10-P90) for each year after BVDV introduction by selecting only repetitions in which the virus was still present in the herd at the end of the year, i.e. at weaning. Virus persistence was calculated weekly.

### Impact of the herd structure outdoors on BVDV spread

To test the effect of our assumptions regarding the structure of herds during the indoor and outdoor periods, two options were compared with the reference scenario: (1) no structure is considered, assuming all animals are homogeneously mixed as indoors; (2) three groups are considered as in the reference case but with no contact between them (β^*P*^_*b*_ = 0).

### Sensitivity analysis

To identify the parameters which influence BVDV spread and its impact on herd productivity, we carried out a sensitivity analysis of the model, assuming here the outdoor reference structure (3 groups with between-group contacts). The selected input parameters were the following:

- parameters related to the herd management: level of numerical productivity expected by the farmer *ε*, duration of both the breeding (*breeding*_*dur*) and outdoor (*pasture*_*dur*) periods, renewal rate of breeding females (*renewal*_*rate*), herd size (*size*), and period of selling (*sell*_*period*);
- parameters related to the infection dynamics: mortality at birth of PI animals (*μ*_*P*,*bi*_), mortality of PI animals (*μ*_*P*_), transition rates (*ϕ*_*MS*_) and (*ϕ*_*TR*_), transmission rates (*β*^*P*^, *β*^*T*^, ${\beta}_b^P$), abortion probabilities in early (*α*_*Ra*_) and mid-pregnancy (*α*_*Rb*_), probability of giving birth to a PI calf for a dam infected during mid-pregnancy (*η*_*P*_), and type of virus introduction (*intro*_*week*).

Three values were tested per parameter (Table 1). For continuous parameters (rates and proportions), we tested variations of 90%, 100%, and 110% of their nominal value (except for *ε* for which the variation was ± 5% to remain within a plausible range, and for η_*P*_ which cannot be above 1). For other parameters (periods, herd size, and virus introduction), we tested for plausible values. Three selling periods are possible in the field, animals (including PI) being kept longer or shorter accordingly. We tested for selling all of the sold animals at each of these 3 periods. Durations of the breeding and the pasture periods varied by ± 2 and 3 weeks, respectively. To cover the variety of herd sizes in the Bourgogne region, we tested three numbers of females kept for breeding: 42 (10 heifers - 32 cows), 83 (20 heifers - 63 cows, reference scenario), and 125 (30 heifers - 95 cows). Finally, BVDV introduction may occur through different ways in a naive beef cattle herd. In addition to the birth of a single PI (week 27, reference scenario), we tested the case of multiple births of PI calves (weeks 20, 25, and 30 successively, i.e. at the start, in mid, and at the end of the calving period), and the case where a PI replacement calf is purchased during the outdoor period (week 40). Other purchased animals could also be infected and therefore introduce BVDV in the herd. However, pregnant females are purchased at the start of the building period, thus when most of the females are in late gestation. Introducing a transiently infected female will barely have any effect. Introducing an immune dam carrying a PI fetus will have the same influence as introducing a PI calf at birth. Introducing a PI pregnant female is quite rare. Lastly introducing a PI bull could have a large effect but only if introduced directly in a group of bred females among which some are already pregnant, which also corresponds to the period of purchase of replacement calves. Therefore, we chose to present here the most probable cases that are the birth of PI calves and the purchase of a PI replacement calf.

Since herd size and the type of virus introduction in the herd were expected to largely impact model outputs, 9 (3 herd sizes × 3 types of introduction) sensitivity analyses were carried out to evaluate the effect of other model parameters. We used a fractional factorial design to sample parameter values [33]. A factorial design is appropriate when the levels of some input variables are discrete (such as periods). In such a design, all the combinations between variable levels are considered, leading to *p*^*n*^ scenarios when *n* parameters with *p* levels are considered. Using a fractional design (using the *proc factex*, SAS) enabled us to considerably reduce the number of scenarios and is appropriate when sensitivity indices for principal effects and first-order interactions only are estimated. Two thousand one-hundred and eighty-seven scenarios were run for each analysis.

We analyzed aggregated outputs calculated as the mean values over the first 5 years after BVDV introduction of *losses* (mortality and abortion) and of the prevalence of T (*prevT*) and PI (*prevP*) animals, and of immune dams carrying a PI fetus (*prevRP*), in an infected herd.

For each output *k*, a linear regression model (ANOVA) was run with all model parameters: *k*_*ij* …_ = *μ* + *f*(*i*, *j*, …) + *ϵ*, with μ a constant, *f* the relation between factors (*i*, *j*, …), and *ϵ* the residual. The total sum of squares then writes: $S{S}_{tot}^k={\displaystyle \sum_{i,j,\dots }}{\left({k}_{ij\dots }-k'\dots \right)}^2=S{S}_i^k+S{S}_j^k+S{S}_{i:j}^k+S{S}_{\in}^k$ (here for two factors *i* and *j*), with $S{S}_i^k$ and $S{S}_{i:j}^k$ the sum of squares related to factor *i* and to the first-order interaction between factors *i* and *j* for output *k*, respectively. The contribution of factor *i* to variations in output *k* is ${C}_i^k=\frac{S{S}_i^k+\frac{1}{2}{\displaystyle {\sum}_{j\ne i}}S{S}_{i:j}^k}{S{S}_{tot}^k}$. The sum of the contributions was equal to model *R*^2^.

### Impact of BVDV spread in an endemic situation

Five years after BVDV first introduction, if the virus is still present in the herd then an endemic state has been reached. To evaluate the impact of BVDV spread in such an endemic situation, cumulated outputs over 10 years (year 6 to 15 after virus introduction) were calculated to enable a comparison between the endemic situation and the acute one (based on the first 3 years). Only repetitions for which virus was present at least one week were included. Moreover, cumulated outputs were normalized to account for the proportion of time the herd truly was infected. Outputs thus were multiplied by the ratio of the number of weeks the virus was present in the herd over the total duration in weeks of the acute and the endemic periods, respectively. The comparison between the acute and the endemic periods was initially evaluated without allowing virus reintroduction through purchases of infected animals or fenceline contacts with infected neighboring herds during the outdoor period. As these factors can significantly influence disease persistence, we also simulated BVDV reintroduction in the herd accounting for a probability of purchasing infected animals (TI, P or immune dam carrying a PI fetus) and for a probability of fenceline contacts with neighboring infected herds during the outdoor period (*K*_*ext*_). As no information was available on observed within-herd prevalence of BVDV infection in infected herds, we assumed a risk of purchasing infected animals on our best knowledge (1% for TI, 1% for P, and 0.5% for immune dams carrying a PI fetus) and assuming two levels of regional prevalence of infected herds: weak (10%) and strong (50%). For fenceline contacts, we evaluated three levels of external risk: nil (*K*_*ext*_ = 0), weak (*K*_*ext*_ = 0.0025), and strong (*K*_*ext*_ = 0.01). Each case was tested for each of the three herd sizes and type of initial virus introduction which is expected to impact the acute phase.

## Results

### BVDV spread in a naive cow-calf herd

Herd size and the type of initial BVDV introduction in the herd impacted the spread and persistence of BVDV in a naive cow-calf herd.

**Figure 3:**
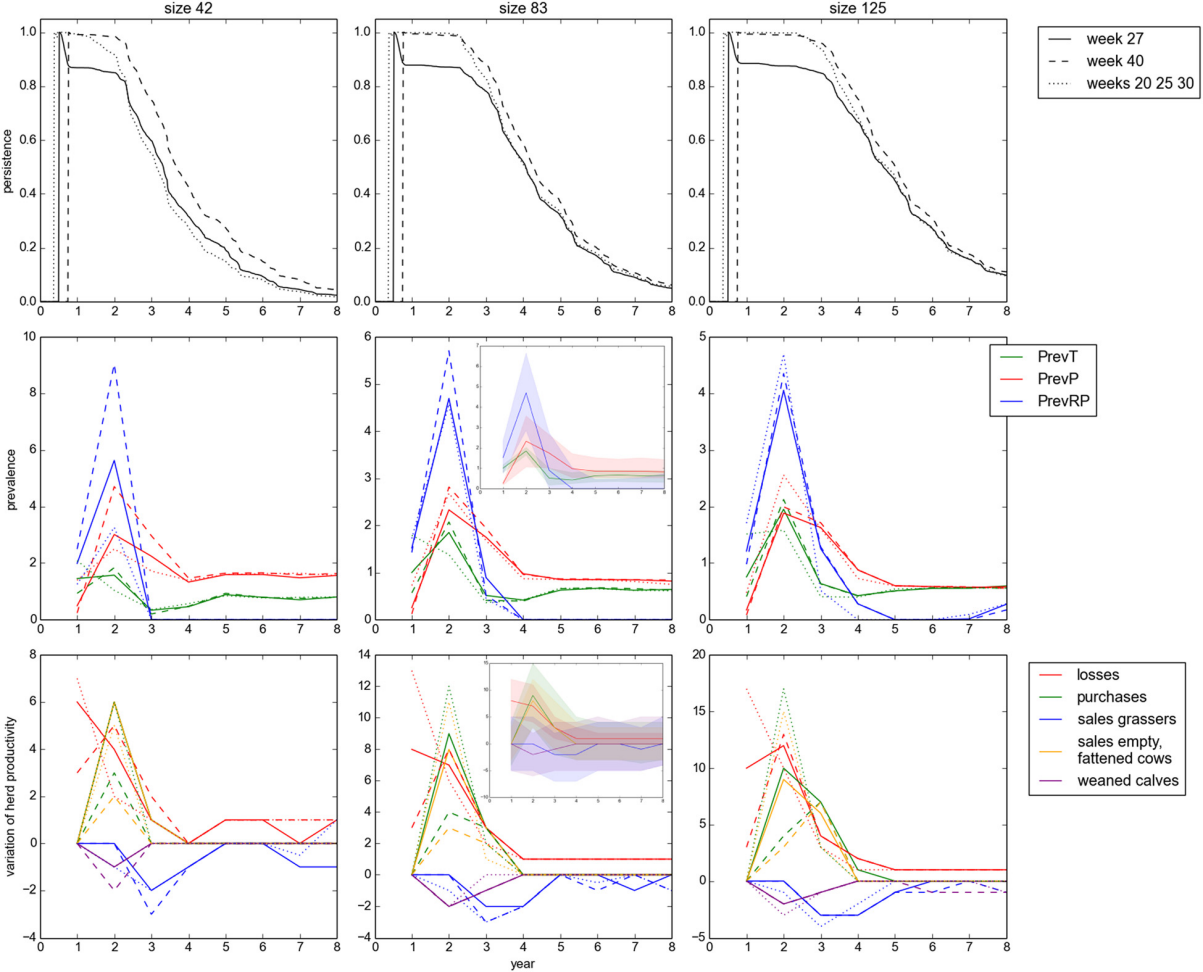
**BVDV spread in a beef cow**-**calf herd according to virus introduction and herd size.** Three types of initial virus introduction were considered: in week 27 (solid line), in week 40 (dashed line), in weeks 20, 25 and 30 (dotted line). Three herd sizes were considered: 42 (on the left), 83 (on the middle), 125 bred females (on the right). The first row shows the probability of virus persistence in the herd over time. The second row shows the median values (with for medium herds an 80% credible interval shown for *intro*_*week* = 27 on the small figure) of the prevalence of transiently (*T*) and persistently infected (PI) animals in the herd, and of immune dams carrying a PI fetus (*RP*) among bred females. The third row shows the median values (with for medium herds an 80% credible interval shown for *intro*_*week* = 27 on the small figure) of productivity outputs: losses (red), purchases (green), sales of grassers and heifers (blue), sales of empty and fattened females (orange), and number of weaned calves (purple). Annual prevalence and productivity outputs were estimated each year considering only repetitions with the virus still present at the end of the year.

Regardless of herd size, introducing the BVDV through multiple births of PI calves (weeks 20, 25, and 30) or through the purchase of a PI replacement calf (week 40) gave rise to virus persistence in almost all of the repetitions for 1 to 3 years, whereas the birth of a single PI (week 27) was followed quickly by a 10% drop in persistence (in 10% of the repetitions, the infection had faded out; Figure 3A). Two years after the initial virus introduction, the persistence reached almost the same level in the three scenarios. Persistence increased with herd size. To reach a 50% probability of BVDV extinction, 3.1 to 3.7 years were needed in small herds, 4 to 4.3 in medium ones, and 4.7 to 5 in large ones. Without reintroduction, the virus persisted for more than 3 years in 55-75%, 78-88%, and 85-96% of the repetitions in small, medium, and large herds, respectively. It persisted for 8 years in 2-4% of the repetitions in small herds vs. 9-11% in large ones.

Regardless of herd size, the annual prevalence of PI animals and immune dams carrying a PI fetus in an infected herd reached a maximum the year after the year of virus introduction (i.e. year 2). The prevalence of transiently infected animals was the highest during the year of virus introduction (year 1) when 3 PI were successively introduced. In the other scenarios, it was the highest the second year. In medium herds, after the birth of a single PI calf, the prevalence on year 2 of T, PI and dams carrying a PI fetus in 80% of the repetitions ranged from 1.6 to 2.0%, 1.0 to 3.6%, and 2.8 to 6.6%, respectively (Figure 3B). The prevalences slightly decreased with herd size. In small herds only, the median prevalences were higher if a PI calf was introduced outdoors (1.8%, 4.7%, 9.0%, respectively) than during the calving period (1.6%, 3.0%, 5.6%, respectively). For other herd sizes, the prevalences were mostly similar among types of virus introduction. Three to four years after BVDV introduction, the prevalences tended to stable values, denoting that an endemic state had been reached which was not affected by the initial virus introduction. An endemically infected herd was predicted to have from 0.3 to 1.1% of T animals, from 0.3 to 2.4% of PI animals, and from none to 0.9% of its dams carrying a PI fetus.

Regardless of herd size and type of virus introduction, the highest impact of BVDV spread on herd productivity occurred during the second year (Figure 3C). Losses associated with abortions and PI mortality were the highest the first two years, ranging from 0.16 to 0.26 per bred female. Losses per bred female were slightly lower in large herds. Additional purchases and sales of empty and fattened females, and losses in weaned calves that were due to BVDV spread were the highest the second year and then rapidly decreased. Purchases and sales of empty and fattened females per bred female were closely related and ranged from 0.05 to 0.17, with no effect of herd size. In more than half the repetitions (median), less than 2 weaned calves were lost due to BVDV spread over 3 years. The number of grassers and heifers sold was not impacted the first two years, a decrease occurring only in the third and fourth years. If the virus was introduced outdoors, losses were later and lower. Without any subsequent virus introduction, the productivity outputs tended to be barely affected 5 years after the first BVDV introduction.

### Impact on BVDV spread of the herd structure outdoors

Assuming a heterogeneous mixing outdoors modified the predicted model outputs compared with assuming a homogeneous mixing. On the contrary, in the case of a heterogeneous mixing outdoors, assuming no contact between groups did not change model predictions compared with assuming the occurrence of between-group contacts (reference scenario).

**Figure 4:**
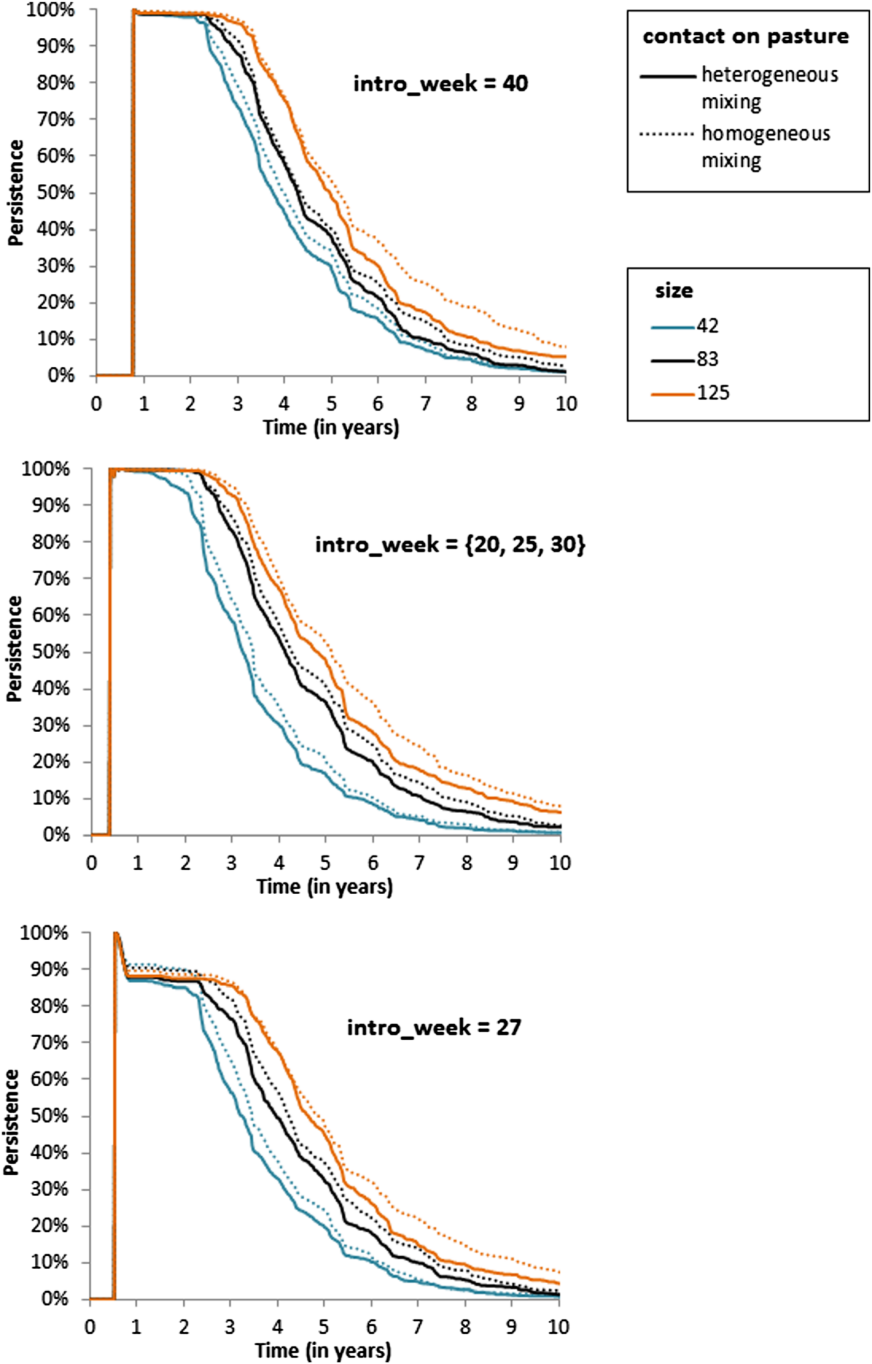
**BVDV persistence in a beef cow**-**calf herd for heterogeneous versus homogeneous contact structure on pasture.** Two scenarios of contact structure were considered (solid line: heterogeneous; dotted line: homogeneous) for three herd sizes (blue: 42 bred females; black: 83; orange: 125) and three types of initial virus introduction (top: in week 40; middle: in weeks 20, 25 and 30; bottom: in week 27).

The predicted virus persistence slightly increased when assuming a homogeneous mixing compared with a heterogeneous one, especially for large herds and for risky types of virus introduction (a PI replacement calf purchased during the outdoor period or 3 PI calves born successively; Figure 4). In the reference scenario, the impact of herd structure can hardly be seen. However, in the largest herds (125 bred females), the virus persisted 8 years after its introduction in 16-18% of the repetitions in the homogeneous mixing scenario compared with 9-12% of the repetitions in the heterogeneous mixing scenario.

The predicted prevalences of PI animals and immune dams carrying a PI fetus in infected herds were higher during the acute phase when assuming a homogeneous vs. heterogeneous mixing outdoors, regardless of herd size and type of virus introduction. In a herd of 83 bred females, the predicted median prevalence of PI animals on year 2 varied with the type of BVDV introduction and ranged from 3.1 to 3.8% in the homogeneous mixing scenario compared with 2.3 to 2.8% in the heterogeneous mixing scenario. The median prevalence of dams carrying a PI fetus on year 2 ranged from 6.0 to 6.7% in the homogeneous mixing scenario compared with 4.6 to 5.7% in the heterogeneous mixing scenario. Herd structure on pasture had no effect of the prevalence of T animals.

Predicted losses (abortion and PI mortality), purchases, and sales of empty and fattened females were slightly higher during the acute phase when assuming a homogeneous mixing outdoors, regardless of herd size and type of virus introduction.

### Impact of herd management and infection characteristics on BVDV spread

**Figure 5:**
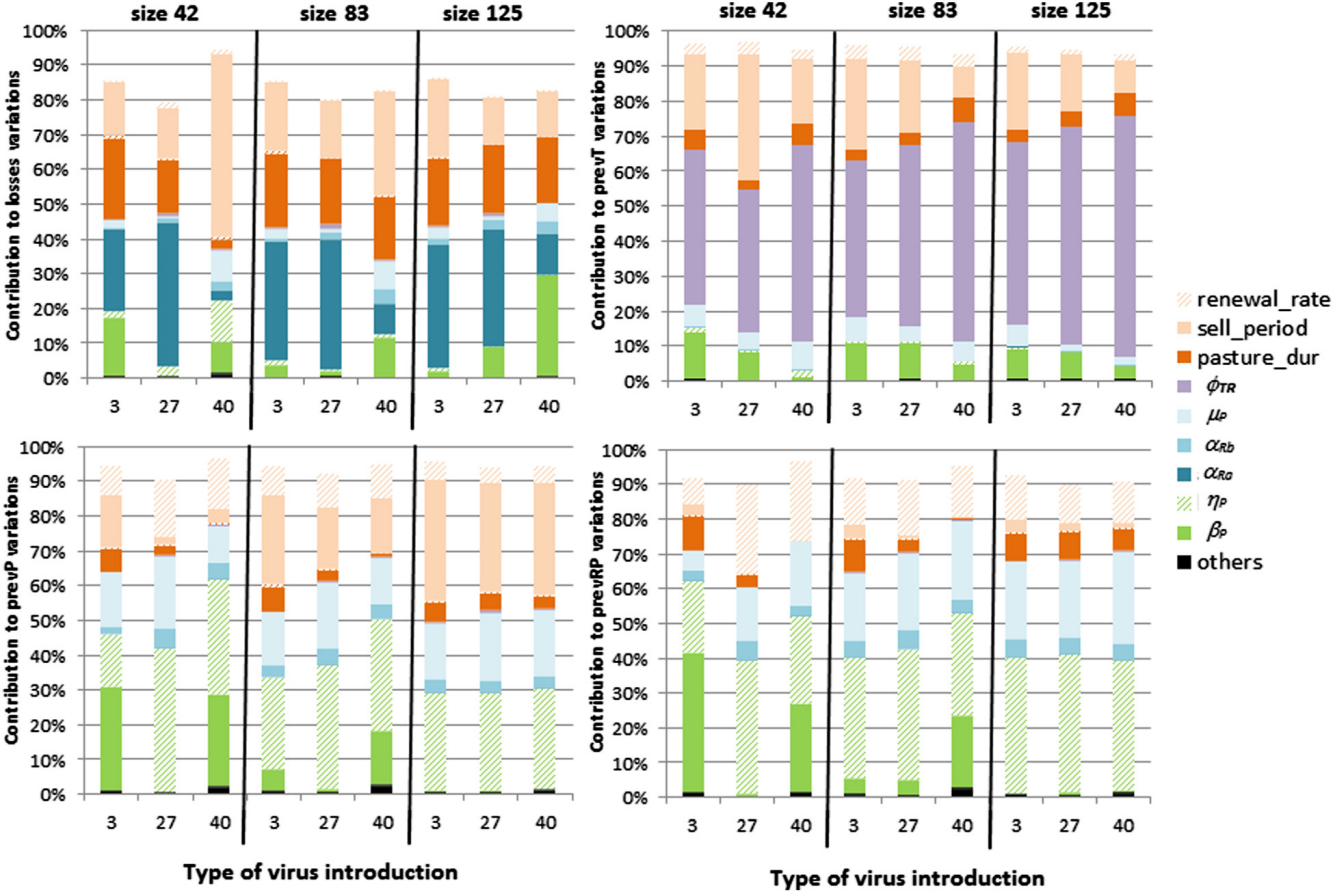
**Total sensitivity indices related to model parameters contributing to output variations.** Sensitivity analyses were done for three herd sizes (42, 83, or 125 bred females) and three types of initial virus introduction (3: in weeks 20, 25 and 30; 27/40: on that specific week). Four aggregated outputs over the first five years were analyzed: losses (top left), prevalence in T animals “prevT” (top right), in PI animals “prevP” (bottom left), and in immune dams carrying a PI fetus “prevRP” (bottom right). See Table 1 for definitions and values of the parameters. Parameters slightly accounting for output variance were grouped (in black).

Herd management and infection characteristics both influenced losses over the first five years of infection, as well as the prevalence of T animals, PI animals, and dams carrying a PI fetus (Figure 5). The type of virus introduction impacted parameters identified as key in the sensitivity analyses, especially in small herds. Otherwise, key parameters were nearly the same regardless of herd size.

Losses varied among scenarios of the sensitivity analysis in the ranges 8-15, 13-30, and 18-42 animals in small, medium, and large herds, respectively. The prevalence of T animals varied among scenarios between 0.8 and 1.6%, the prevalence of PI animals varied between 0.9 and 3.0%, and the prevalence of dams carrying a PI fetus varied from 1.4 and 4.8%, irrespective of herd size.

Losses variations were mainly explained by pasture duration (*pasture*_*dur*) and the choice of the selling period (*sell*_*period*) for parameters related to herd management, and by the abortion rate due to infection in early pregnancy (*α*_*Ra*_) and the transmission rate by PI animals ($\beta$^*P*^) for parameters related to infection characteristics (Figure 5A). Introducing a PI replacement calf (week 40) in small herds led to a decrease in the contributions of these key parameters except *sell*_*period*, and led to an additional contribution of the probability of given birth to a PI calf for dams infected in mid-pregnancy (η_*P*_) and of PI mortality (μ_*P*_).

The variations in the prevalence of T animals in an infected herd (Figure 5B) were mainly explained by the choice of the selling period (*sell*_*period*), the transient infection duration (ϕ_*TR*_), and slightly by pasture duration (*pasture*_*dur*), the transmission rate by PI animals (β^*P*^), and PI mortality (μ_*P*_). The variations in the prevalence of PI animals (Figure 5C) were mainly explained by *sell*_*period* and the renewal rate (*renewal*_*rate*), and in some cases *pasture*_*dur*, as well as by the probability of given birth to a PI calf for dams infected in mid-pregnancy (η_*P*_), μ_*P*,_ and in some cases β^*P*^. The variations in the prevalence of dams carrying a PI fetus (Figure 5D) were mainly explained by the same parameters as the variations in the prevalence of PI animals, except *sell*_*period* which barely contributed.

### BVDV spread in an endemic situation

The risk of buying infected animals was low. Even when half of the source herds for replacement animals were assumed to be infected with BVDV, the model predicted that BVDV would be reintroduced to medium herds only once every 20 years during the endemic period. Indeed, only 1 to 5 animals were purchased per year per herd irrespective of the BVDV herd status, with a low risk that these animals were infected due to the low within-herd prevalence of infection.

The tested levels for an external risk of infection on pasture (*K*_*ext*_) corresponded to BVDV reintroductions during the endemic phase once every 6-7 years for the low level, and once every 2-3 years for the high level (Table 2). During the acute phase, as herds were mainly already infected, herd reinfection barely occurred. Median prevalence of infection in infected herds was twice as high during the acute phase as during the endemic phase (Table 2). However, cumulative losses, purchases, sales and variations in weaned calves over 10 years per bred female in an endemic situation greatly surpassed the ones occurring during the acute phase (Table 2). Moreover, when BVDV was reintroduced once every 2-3 years, losses per bred females during the endemic phase in an infected herd were twice as high as when BVDV was not reintroduced or was reintroduced only once every 6-7 years (with for example 36% vs. 15% of losses per bred female in medium herds).

**Table 2:** **Comparison of the outputs of the model of BVDV spread in a beef cow**-**calf herd cumulated over the acute** (**years 1 to 3 after initial introduction**) **versus the endemic phase** (**years 6 to 15**)

**Output definition**	**Acute phase**	**Endemic phase**
Average probability of virus presence^1^	0.75-0.76-0.76	0.14-0.17-0.25
Average frequency of herd reinfection^1^ (yr^-1^)	0-0.02-0.08	0-0.14-0.38
Median prevT (when the virus is present) (%)	1.3-1.5	0.5-1.0
Median prevP (when the virus is present) (%)	1.4-3.5	0-1.2
Median prevRP (when the virus is present) (%)	2.1-5.5	0-1.3
Median losses/100 bred females	23-37	12-53
Median purchases/100 bred females	13-26	34-125
Median sales of grassers & heifers/100 bred females	-7--2	-1-29
Median sales of fattened females/100 bred females	10-23	23-108
Median weaned calves/100 bred females	-5--2	2-39

Ranges came from variations in herd size, type of initial virus introduction, and external risk of reintroduction.

^1^For three levels of *K*_*ext*_ = {0, 0.0025, 0.01} (per day).

## Discussion

We propose a stochastic model of BVDV spread in a structured beef cow-calf cattle herd. Originally, we accounted for a variation in exposure of animals (especially adults) between the indoor period, during which a homogeneous contact structure is assumed, and the outdoor period, during which groups are formed and raised on different pastures. Moreover, we accounted for a risk of continuous virus introduction in the herd through animal purchases and contacts with neighboring infected herds. This enabled the model to represent a large range of possible situations, from a single introduction into a naive herd to endemic situations potentially maintained by an external risk of virus reintroduction. Lastly, the model was flexible enough to represent beef herds of different sizes and management types, as illustrated by the different types of herds observed in Bourgogne, one of the main beef cattle farming regions in France. Such a model was pertinent to investigate the impact of BVDV spread on the productivity of a herd for a large range of scenarios.

To precisely represent the infection process, several modelling choices had to be made. The model was stochastic to enable an estimation of the probability of virus persistence. The variability in model outputs if the virus persisted was not very large. However, the prevalence in PI animals and in immune dams carrying a PI fetus in infected herds was low and therefore better estimated using a stochastic model. A discrete time step of 1 week was used as the longest permitting to precisely estimate the morbidity related to transiently infected animals (transient infection being quite short). We chose to implement a combination of compartmental and individual-based models - instead of using a fully individual-based approach as used in [34,35] – with the anticipation of using this within-herd model as part of a more complex simulation framework where computational efficiency is paramount, to represent between-herd BVDV spread and control at a regional scale. Indeed, representing individually each animal all its lifetime was not necessary, except during pregnancy to precisely predict when infection will occur relative to the stage of pregnancy of the dam, and which consequence it will have for the calf to be born. Hence, we integrated in the compartmental model an individual-based monitoring of pregnant females (from the start of pregnancy until calving). To account for the seasonality of breeding and outdoor contacts, discrete periods were designed for reproduction and grazing. Such an approach is suitable when the continuous epidemic process (infection occurring possibly each day) is influenced by seasonal population dynamics (and therefore seasonal contacts) occurring on a discrete basis (with for instance only two periods in a year with different types or levels of contact). All of the model parameters related to herd size (number of bred cows and heifers) and management (target number of weaned calves, occurrence and intensity of purchases and of neighboring relationships, within herd contact structure, breeding/calving and indoor/outdoor periods) are user-defined. Hence, our model is highly flexible and may also be used to represent beef cattle herds in other regions.

The simulation results show that failing to account for the separation of cattle into different management groups on pasture could lead to overestimations of the predicted disease prevalence and persistence in affected herds. In our study, we assumed that there were three management groups and that these management groups remained fixed over the entire grazing period. However, in the real world, herd structure is often dictated by complex management constraints such as herd size, pasture availability, and labor resources. Furthermore, the management groups of bred females with bulls may be reformed several times during the grazing period, which may lead towards more homogeneous mixing dynamics. There is a need for further research into the effects of herd structure on BVDV spread using empirically derived data.

When introduced into a naive herd, BVDV spread was shown to have a large impact on herd productivity, especially the first 3 years after the initial introduction of the virus, during which yearly losses may be up to 6 times higher than in subsequent years when herd immunity has developed. The impact expressed per bred female was slightly lower in larger herds. Moreover, in the absence of control measures, BVDV may persist for years, which is currently observed in some regions [3,4,36,37]. We found that the virus was more likely to persist over time in larger herds. Virus persistence may increase with herd size because self-clearance may be more frequent in small herds due to stochastic events [38,39]. In such an endemic situation, our model predicted that yearly losses will be limited and that the production objective in weaned calves will be reached most of the time, in good agreement with the results obtained for Scottish beef herds [5]. However, the low yearly losses have to be balanced by the duration of virus persistence. Cumulated over several years, losses occurring during the endemic phase cannot be neglected, especially when virus reintroduction is frequent.

The sensitivity analysis shows that herd management (through pasture duration, selling period, and renewal rate) and the infection dynamics (though the transmission rate from PI, the duration of transient infection, the vertical transmission, PI mortality, and abortion rates) were very influential processes on at least some of the model outputs (prevalence of T and PI animals and of immune dams carrying a PI fetus, losses), regardless of herd size and type of virus introduction. As for dairy herds [10], it suggests that control of PI animals is the key for preventing BVDV spread in beef herds. PI animals may be introduced via either purchases or births to immune dams infected during mid-pregnancy. In Bourgogne, the breeding period occurs essentially during the outdoor period. Hence, it is an at-risk period for breeding females since they can become infected from contact with neighboring herds.

Among the available strategies to control BVDV spread in beef herds, vaccination is one of the main current options. According to our findings, female vaccination before breeding seems to be a valuable strategy to limit losses due to BVDV spread and persistence [40-43]. Vaccination has been used largely in the US [44], but much less in the EU [43], except in Germany where it has been used in combination with eradication [45]. Such a strategy could become efficient and should be further evaluated in a context where herds may be regularly reinfected through neighboring contact. In the present study, the impact of BVDV on herd productivity was evaluated by measuring biological outputs (losses, variations in weaned calves, etc.). For the model to be useful in evaluating control strategies, it would be important to assign economic values to variations in herd productivity [5,18] and to account for farmer's decisions [46,47]. Indeed, farmers may be unwilling to implement control measures if the economic impact of BVDV on their herd is perceived to be low. Farmers of endemically infected herds may also become unaware of their own BVDV status [3,4,36] and of the risk they pose of transmitting the virus through animal movements and neighboring contact with other herds [48]. Moreover, if the risk of disease reintroduction is high, farmers may not perceive the value of controlling it even if they are aware it is present. Our model then can be used to estimate the expected prevalence of PI animals as well as of immune dams carrying a PI fetus in such endemically infected herds, therefore providing a prior for the risk of infection for (potentially naive) contact herds. Several groups are identified in the modelled herd based on age/physiological stages and health statuses, enabling the model to be used in the future to evaluate targeted control strategies at the herd scale in either naive or endemic situations.

## References

[1] Stott AW, Humphry RW, Gunn GJ (2010). Modelling the effects of previous infection and re-infection on the costs of bovine viral diarrhoea outbreaks in beef herds. Vet J.

[2] Joly A, Fourichon C, Beaudeau F (2005). Description and first results of a BVDV control scheme in Brittany (western France). Prev Vet Med.

[3] Gates MC, Woolhouse MEJ, Gunn GJ, Humphry RW (2013). Relative associations of cattle movements, local spread, and biosecurity with bovine viral diarrhoea virus (BVDV) seropositivity in beef and dairy herds. Prev Vet Med.

[4] Gates MC, Humphry RW, Gunn GJ (2013). Associations between bovine viral diarrhoea virus (BVDV) seropositivity and performance indicators in beef suckler and dairy herds. Vet J.

[5] McCormick B, Stott A, Brülisauer F, Vosough Ahmadi B, Gunn G (2010). An integrated approach to assessing the viability of eradicating BVD in Scottish beef suckler herds. Vet Microbiol.

[6] Bennett RM (1992). Case-study of a simple decision support system to aid livestock disease control decisions. Agr Syst.

[7] Ezanno P, Fourichon C, Seegers H (2008). Influence of herd structure and type of virus introduction on the spread of bovine viral diarrhoea virus (BVDV) within a dairy herd. Vet Res.

[8] Ezanno P, Vergu E, Langlais M, Gilot-Fromont E: Modelling the dynamics of host-parasite interactions: basic principles. In New Frontiers of Molecular Epidemiology of Infectious Diseases. Edited by Morand S, Beaudeau F, Cabaret J: Springer; 2012:79-101.

[9] Viet A-F, Fourichon C, Seegers H (2007). Review and critical discussion of assumptions and modelling options to study the spread of the bovine viral diarrhoea virus (BVDV) within a cattle herd. Epidemiol Infect.

[10] Ezanno P, Fourichon C, Viet A-F, Seegers H (2007). Sensitivity analysis to identify key-parameters in modelling the spread of bovine viral diarrhoea virus in a dairy herd. Prev Vet Med.

[11] Courcoul A, Ezanno P (2010). Modelling the spread of Bovine Viral Diarrhoea Virus (BVDV) in a managed metapopulation of cattle herds. Vet Microbiol.

[12] Ersbøll AK, Ersbøll BK, Houe H, Alban L, Kjeldsen A (2010). Spatial modelling of the between-herd infection dynamics of bovine virus diarrhoea virus (BVDV) in dairy herds in Denmark. Prev Vet Med.

[13] Tinsley M, Lewis FI, Brülisauer F (2012). Network modeling of BVD transmission. Vet Res.

[14] Houe H (1993). Survivorship of animals persistently infected with bovine virus diarrhoea virus (BVDV). Prev Vet Med.

[15] Gunn GJ, Stott AW, Humphry RW (2004). Modelling and costing BVD outbreaks in beef herds. Vet J.

[16] Smith RL, Sanderson MW, Renter DG, Larson R, White B (2010). A stochastic risk-analysis model for the spread of bovine viral diarrhea virus after introduction to naive cow-calf herds. Prev Vet Med.

[17] Nickell JS, White BJ, Larson RL, Renter DG, Sanderson MW (2011). A simulation model to quantify the value of implementing whole-herd Bovine viral diarrhea virus testing strategies in beef cow-calf herds. J Vet Diagn Invest.

[18] Smith RL, Sanderson MW, Jones R, N’Guessan Y, Renter D, Larson R, White BJ (2013). Economic risk analysis model for bovine viral diarrhea virus biosecurity in cow-calf herds. Prev Vet Med.

[19] Viet A-F, Ezanno P, Petit E, Devun J, Vermesse R, Fourichon C (2012). Resilience of a beef cow-calf farming system to variations in demographic parameters. J Anim Sci.

[20] Lindberg ALE (2003). Bovine viral diarrhoea virus infections and its control. A review Vet Q.

[21] Kendrick JW (1971). Bovine viral diarrhea-mucosal disease virus infection in pregnant cows. Am J Vet Res.

[22] Done J, Terlecki S, Richardson C, Harkness J, Sands J, Patterson D, Sweasey D, Shaw I, Winkler C, Duffell S (1980). Bovine virus diarrhoea-mucosal disease virus: pathogenicity for the fetal calf following maternal infection. Vet Rec.

[23] McClurkin AW, Littledike ET, Cutlip RC, Frank GH, Coria MF, Bolin SR (1984). Production of cattle immunotolerant to bovine viral diarrhea virus. Can J Comp Med.

[24] Carlsson U, Fredriksson G, Alenius S, Kindahl H (1989). Bovine virus diarrhoea virus, a cause of early pregnancy failure in the cow. Zentralbl Veterinarmed A.

[25] McGowan M, Kirkland P, Richards S, Littlejohns I (1993). Increased reproductive losses in cattle infected with bovine pestivirus around the time of insemination. Vet Rec.

[26] Moerman A, Straver P, de Jong M, Quak J, Baanvinger T, van Oirschot J (1993). A long term epidemiological study of bovine viral diarrhoea infections in a large herd of dairy cattle. Vet Rec.

[27] Breto C, He D, Ionides EL, King AA (2009). Time series analysis via mechanistic models. Ann Appl Stat.

[28] Saltelli A, Chan K, Scott EM (2000). Sensitivity Analysis.

[29] Viet A-F, Fourichon C, Seegers H, Jacob C, Guihenneuc-Jouyaux C (2004). A model of the spread of the bovine viral-diarrhoea virus within a dairy herd. Prev Vet Med.

[30] Carslake D, Grant W, Green LE, Cave J, Greaves J, Keeling M, McEldowney J, Weldegebriel H, Medley GF (2011). Endemic cattle diseases: comparative epidemiology and governance. Philos Trans R Soc Lond B Biol Sci.

[31] Sarrazin S, Veldhuis A, Méroc E, Vangeel I, Laureyns J, Dewulf J, Van Der Stede Y (2013). Serological and virological BVDV prevalence and risk factor analysis for herds to be BVDV seropositive in Belgian cattle herds. Prev Vet Med.

[32] Moennig V, Houe H, Lindberg A (2005). BVD control in Europe: current status and perspectives. Anim Health Res Rev.

[33] Ståhl K, Lindberg A, Rivera H, Ortiz C, Moreno-López J (2008). Self-clearance from BVDV infections—a frequent finding in dairy herds in an endemically infected region in Peru. Prev Vet Med.

[34] Lindberg AL, Alenius S (1999). Principles for eradication of bovine viral diarrhoea virus (BVDV) infections in cattle populations. Vet Microbiol.

[35] Greiser-Wilke I, Grummer B, Moennig V (2003). Bovine viral diarrhoea eradication and control programmes in Europe. Biologicals.

[36] Moennig V, Brownlie J: Vaccines and vaccination strategies. In EU Thematic network on control of bovine viral diarrhoea virus: position paper (QLRT – 2001-01573). Edited online (http://www.afbini.gov.uk/chs-thematic-network-position-paper-on-bvd-control.pdf); 2006:73-98.

[37] Lindberg A, Brownlie J, Gunn G, Houe H, Moennig V, Saatkamp H, Sandvik T, Valle P (2006). The control of bovine viral diarrhoea virus in Europe: today and in the future. Rev Sci Tech.

[38] Ståhl K, Alenius S (2012). BVDV control and eradication in Europe - an update. Jpn J Vet Res.

[39] Fulton RW (2013). Host response to bovine viral diarrhea virus and interactions with infectious agents in the feedlot and breeding herd. Biologicals.

[40] Moennig V, Eicken K, Flebbe U, Frey HR, Grummer B, Haas L, Greiser-Wilke I, Liess B (2005). Implementation of two-step vaccination in the control of bovine viral diarrhoea (BVD). Prev Vet Med.

[41] Rat-Aspert O, Fourichon C (2010). Modelling collective effectiveness of voluntary vaccination with and without incentives. Prev Vet Med.

[42] Santarossa J, Stott A, Humphry R, Gunn G (2005). Optimal risk management versus willingness to pay for BVDV control options. Prev Vet Med.

[43] Bitsch V, Hansen KE, Rønsholt L (2000). Experiences from the Danish programme for eradication of bovine virus diarrhoea (BVD) 1994–1998 with special reference to legislation and causes of infection. Vet Microbiol.

[44] Duffell S, Harkness J (1985). Bovine virus diarrhoea-mucosal disease infection in cattle. Vet Rec.

[45] Kendrick JW, Franti CE (1974). Bovine viral diarrhea: decay of colostrum-conferred antibody in the calf. Am J Vet Res.

[46] Baker JC (1987). Bovine viral diarrhoea virus: a review. J Am Vet Med Assoc.

[47] Mars MH, Bruschke CJM, van Oirschot JT (1999). Airborne transmission of BHV1, BRSV, and BVDV among cattle is possible under experimental conditions. Vet Microbiol.

[48] Niskanen R, Lindberg A (2003). Transmission of bovine viral diarrhoea virus by unhygienic vaccination procedures, ambient air, and from contaminated pens. Vet J.

